# Genome Editing Method for the Anaerobic Magnetotactic Bacterium Desulfovibrio magneticus RS-1

**DOI:** 10.1128/AEM.01724-18

**Published:** 2018-10-30

**Authors:** Carly R. Grant, Lilah Rahn-Lee, Kristen N. LeGault, Arash Komeili

**Affiliations:** a Department of Plant and Microbial Biology, University of California, Berkeley, Berkeley, California, USA; University of Tartu

**Keywords:** Desulfovibrio, biomineralization, genome editing, iron, magnetosomes, magnetotactic bacteria, organelles

## Abstract

Magnetosomes are complex bacterial organelles that serve as model systems for studying bacterial cell biology, biomineralization, and global iron cycling. Magnetosome biogenesis is primarily studied in two closely related Alphaproteobacteria of the genus Magnetospirillum that form cubooctahedral-shaped magnetite crystals within a lipid membrane. However, chemically and structurally distinct magnetic particles have been found in physiologically and phylogenetically diverse bacteria. Due to a lack of molecular genetic tools, the mechanistic diversity of magnetosome formation remains poorly understood. Desulfovibrio magneticus RS-1 is an anaerobic sulfate-reducing deltaproteobacterium that forms bullet-shaped magnetite crystals. A recent forward genetic screen identified 10 genes in the conserved magnetosome gene island of D. magneticus that are essential for its magnetic phenotype. However, this screen likely missed mutants with defects in crystal size, shape, and arrangement. Reverse genetics to target the remaining putative magnetosome genes using standard genetic methods of suicide vector integration have not been feasible due to the low transconjugation efficiency. Here, we present a reverse genetic method for targeted mutagenesis in D. magneticus using a replicative plasmid. To test this method, we generated a mutant resistant to 5-fluorouracil by making a markerless deletion of the *upp* gene that encodes uracil phosphoribosyltransferase. We also used this method for targeted marker exchange mutagenesis by replacing *kupM*, a gene identified in our previous screen as a magnetosome formation factor, with a streptomycin resistance cassette. Overall, our results show that targeted mutagenesis using a replicative plasmid is effective in D. magneticus and may also be applied to other genetically recalcitrant bacteria.

**IMPORTANCE** Magnetotactic bacteria (MTB) are a group of organisms that form intracellular nanometer-scale magnetic crystals though a complex process involving lipid and protein scaffolds. These magnetic crystals and their lipid membranes, termed magnetosomes, are model systems for studying bacterial cell biology and biomineralization and are potential platforms for biotechnological applications. Due to a lack of genetic tools and unculturable representatives, the mechanisms of magnetosome formation in phylogenetically deeply branching MTB remain unknown. These MTB contain elongated bullet-/tooth-shaped magnetite and greigite crystals that likely form in a manner distinct from that of the cubooctahedral-shaped magnetite crystals of the genetically tractable MTB within the Alphaproteobacteria. Here, we present a method for genome editing in Desulfovibrio magneticus RS-1, a cultured representative of the deeply branching MTB of the class Deltaproteobacteria. This marks a crucial step in developing D. magneticus as a model for studying diverse mechanisms of magnetic particle formation by MTB.

## Abstract (precis)

Magnetotactic bacteria (MTB) are a group of organisms that form intracellular nanometer-scale magnetic crystals though a complex process involving lipid and protein scaffolds. These magnetic crystals and their lipid membranes, termed magnetosomes, are model systems for studying bacterial cell biology and biomineralization and are potential platforms for biotechnological applications. Due to a lack of genetic tools and unculturable representatives, the mechanisms of magnetosome formation in phylogenetically deeply branching MTB remain unknown. These MTB contain elongated bullet-/tooth-shaped magnetite and greigite crystals that likely form in a manner distinct from that of the cubooctahedral-shaped magnetite crystals of the genetically tractable MTB within the Alphaproteobacteria. Here, we present a method for genome editing in Desulfovibrio magneticus RS-1, a cultured representative of the deeply branching MTB of the class Deltaproteobacteria. This marks a crucial step in developing D. magneticus as a model for studying diverse mechanisms of magnetic particle formation by MTB.

## INTRODUCTION

Magnetotactic bacteria (MTB) are a group of diverse microorganisms that align along magnetic fields via their intracellular chains of magnetic crystals (1, 2). Each magnetic crystal consists of either magnetite (Fe_3_O_4_) or greigite (Fe_3_S_4_) and is synthesized within a complex organelle called a magnetosome (3). The first cultured MTB were microaerophilic members of the Alphaproteobacteria, which form cubooctahedral-shaped magnetite crystals and have served as model organisms for understanding magnetosome formation (4–7). Early studies on Magnetospirillum spp. revealed a lipid-bilayer membrane, with a unique suite of proteins, surrounding each magnetite crystal (8–10). The development of genetic tools in Magnetospirillum magneticum AMB-1 and Magnetospirillum gryphiswaldense MSR-1 revealed a conserved magnetosome gene island (MAI) that contains the factors necessary and sufficient for the formation of the magnetosome membrane, magnetite biomineralization within the lumen of the magnetosome, and alignment of the magnetosomes in a chain along the length of the cell (3, 11). These molecular advances, along with the magnetic properties of magnetosomes, have made MTB ideal models for the study of compartmentalization and biomineralization in bacteria as well as a target for the development of biomedical and industrial applications.

Improvements in isolation techniques and sequencing have revealed that MTB are ubiquitous in many aquatic environments. On the basis of phylogeny and magnetosome morphology, MTB can be categorized into two subgroups. The first subgroup includes members of the Alphaproteobacteria and Gammaproteobacteria, such as Magnetospirillum spp., that synthesize cubooctahedral, elongated octahedral, or elongated prisms of magnetite (12). The second subgroup comprises MTB from more deep-branching lineages, including members of the Deltaproteobacteria class and the Nitrospirae and Omnitrophica phyla, which synthesize elongated bullet-/tooth-shaped magnetite and/or greigite crystals (13, 14). While all MTB sequenced to date have their putative magnetosome genes arranged in distinct regions of their genomes (3, 15–17), many of the genes essential for magnetosome biogenesis in Magnetospirillum spp. are missing from the genomes of deep-branching MTB (14). Likewise, a conserved set of *mad* (magnetosome-associated *Deltaproteobacteria*) genes are only found in deep-branching MTB (14, 18–20). This suggests a genetic diversity underpinning the control of magnetosome morphology and physiology in nonmodel MTB that is distinct from that of the well-characterized Magnetospirillum spp.

Desulfovibrio magneticus RS-1, one of the few cultured MTB outside the Alphaproteobacteria, is an anaerobic sulfate-reducing member of the Deltaproteobacteria that forms irregular bullet-shaped crystals of magnetite (21, 22). As with the Magnetospirillum spp., the magnetosome genes of D. magneticus are located within a MAI and include homologs to some *mam* genes as well as *mad* genes (14, 18, 23). Recently, we used a forward genetic screen combining random chemical and UV mutagenesis with whole-genome resequencing to identify mutations that resulted in nonmagnetic phenotypes. These included many mutants that had the entire MAI deleted (ΔMAI) as well as mutants with point mutations, frameshift mutations, and transposon insertions in 10 *mam* and *mad* genes of the D. magneticus MAI that resulted in nonmagnetic phenotypes (20). However, this screen relied on a strict selection scheme for nonmagnetic mutants. As such, we likely missed magnetosome genes that are important for regulating the shape, size, and arrangement of magnetosomes. To elucidate the degree of conservation between *mam* genes and determine the function of the proteins encoded by *mad* genes in D. magneticus, a reverse genetic method for targeted mutagenesis is necessary.

**FIG 1:**
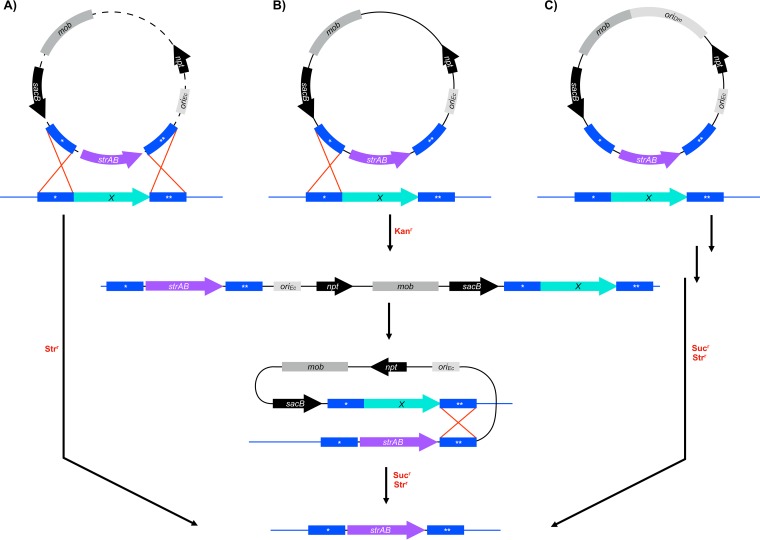
Schematic of deletion methods used in Desulfovibrio spp. Plasmids (black lines) were designed to replace a target gene (*X*, aqua arrows) in the chromosome (blue lines) with a streptomycin resistance cassette (*strAB*, purple arrows). Regions upstream (*) and downstream (**) of the target gene (blue boxes) on the chromosome undergo recombination (red lines) with homologous regions that are cloned into the deletion plasmid. Key steps, such as recombination events (red crosses), are indicated in the boxes, and the selection steps are labeled in red. (A) Double recombination can occur in one step after plasmids are linearized (dashed lines) by endogenous restriction enzymes. Mutants are selected using the marker (e.g., *strAB*) that was exchanged with the target gene. (B) Two-step double recombination is possible when suicide vectors integrate into the chromosome in the first homologous recombination event and then recombine out after the second homologous recombination event. The first step and second step are selected for with antibiotic resistance markers (e.g., *npt*) and counterselectable markers (e.g., *sacB*), respectively. (C) A replicative deletion plasmid designed to target genes for deletion may undergo double recombination in one or two steps as shown in panels A and B, respectively. After passaging the cells without antibiotic, the mutants are selected with an antibiotic resistance cassette (e.g., *strAB*) and a counterselectable marker (e.g., *sacB*). *mob*, mobilization genes (*mobA*′, *mobB*, *mobC*); *npt*, kanamycin-resistance gene; *ori_Dm_*, origin of replication for D. magneticus; *ori_Ec_*, origin of replication for E. coli.

D. magneticus and other Desulfovibrio spp. have gained much attention for their importance in the global cycling of numerous elements, in biocorrosion, and in the bioremediation of toxic metal ions (24, 25). The development of genetic tools, such as expression vectors, transposons, and targeted genome-editing systems, has enabled a more detailed examination of the important activities of a few Desulfovibrio spp. (26, 27). Targeted mutagenesis using a one-step double recombination method was first achieved in Desulfovibrio fructosivorans and, more recently, in Desulfovibrio gigas and Desulfovibrio desulfuricans ND132 (28–30). With this method, plasmids that are electroporated into the cell are thought to be rapidly linearized by endogenous restriction modification systems (30–32). The linearized plasmid DNA, carrying a selectable marker flanked by upstream and downstream regions of homology to a target gene, can then undergo double recombination into the chromosome in one step (Fig. 1A). This efficient one-step method, which is dependent on electroporation of the plasmid (28–30), is unlikely to be applicable for D. magneticus, because plasmid uptake has only been demonstrated using conjugal transfer (20). The second targeted mutagenesis method, used in Desulfovibrio vulgaris Hildenborough, is a two-step double recombination that makes use of a nonreplicative, or suicide, vector (31, 32). In the first step of this method, a suicide vector, with sequences upstream and downstream of the target gene, integrates into the genome upon the first homologous recombination event (Fig. 1B). Next, a second recombination event occurs whereby the vector is excised from the genome, and cells with the desired genotype are selected with an antibiotic marker and/or a counterselection marker (31, 32) (Fig. 1B). For many bacteria, including D. magneticus, plasmid uptake and integration occur at frequencies that are too low for genetic manipulation via suicide vectors (20).

Here, we describe the method we developed for targeted gene deletion using a replicative plasmid, thereby bypassing the need for suicide vector integration (Fig. 1C). We generated a mutant resistant to 5-fluorouracil by making a markerless deletion of the *upp* gene, which encodes an enzyme in the pyrimidine salvage pathway that is nonessential under standard laboratory conditions. Additionally, we deleted *kupM*, a gene encoding a potassium transporter that acts as a magnetosome formation factor (20), via marker exchange with a streptomycin resistance cassette. The deletion of both *upp* and *kupM* conferred the expected phenotypes, which were subsequently complemented in *trans*. Overall, our results show that targeted mutagenesis using a replicative plasmid is possible in D. magneticus. It may also be suitable for other bacteria for which replicative plasmid uptake is possible but at a rate too low for suicide vector integration.

## RESULTS

### Design of a replicative deletion plasmid using *sacB* counterselection.

Targeted genetic manipulation in most bacteria requires a method to efficiently deliver foreign DNA destined for integration into the chromosome. One commonly used method involves suicide vector uptake and integration prior to the first selection step (Fig. 1B). In D. magneticus, plasmid transfer has only been achieved via conjugation at low efficiencies, making the uptake and subsequent integration of suicide vectors into its chromosome an unlikely event (20). As such, we attempted to bypass the use of suicide vectors and use a stable replicative plasmid designed to delete specific genes via homologous recombination (Fig. 1C). Two features of this method enable the isolation of desired mutants: (i) a selectable marker is used to identify double recombination events at the targeted site and (ii) a counterselectable marker distinguishes the desired mutant cells, which have lost all remaining copies of the plasmid.

**FIG 2:**
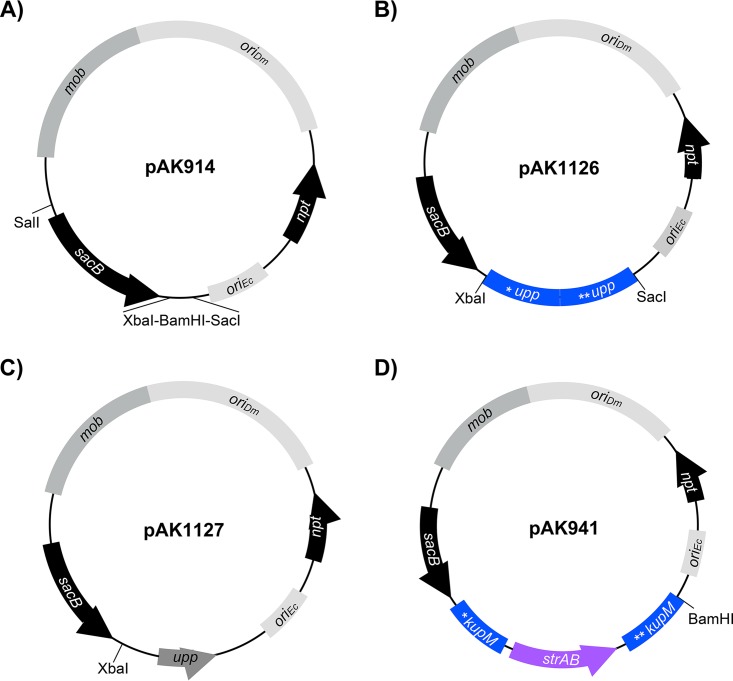
Plasmids constructed for the present study. (A) Expression plasmid pAK914 expresses *sacB* from the *mamA* promoter and is the parent vector for the deletion plasmids and *upp* expression plasmid described below. (B) Replicative deletion plasmid to target *upp* for markerless deletion. The *upp* deletion cassette was cloned into XbaI-SacI of pAK914. (C) Expression plasmid used for *upp* complementation. The *upp* gene and its promoter were cloned into BamHI-SacI of pAK914. (D) Replicative deletion plasmid to target *kupM* for marker exchange mutagenesis with *strAB*. The *kupM*::*strAB* deletion cassette was cloned into XbaI of pAK914. Labeling and colors correspond to those in Fig. 1.

*sacB* is a common counterselection marker that is effective in many bacteria. The *sacB* gene from Bacillus subtilis encodes levansucrase, which converts sucrose to levans that are lethal to many Gram-negative bacteria, including D. vulgaris Hildenborough (31, 33, 34). To test its functionality in D. magneticus, we inserted *sacB* under the expression of the *mamA* promoter of D. magneticus (described in reference 20) in a plasmid that replicates in both Escherichia coli and D. magneticus (Fig. 2A). This plasmid (pAK914) and a control plasmid were then conjugated into D. magneticus. We found no growth inhibition for D. magneticus cells with the control plasmid in the presence of sucrose and kanamycin. In contrast, cells expressing *sacB* were unable to grow with kanamycin and sucrose concentrations of 1% (wt/vol) or higher (data not shown). To test if the plasmids could be cured, D. magneticus with pAK914 was passaged two times in liquid medium containing no antibiotic and plated on 1% sucrose. Individual sucrose-resistant (Suc^r^) colonies were inoculated and screened for kanamycin sensitivity (Kan^s^). All isolated colonies (*n* = 16) were Kan^s^, suggesting that the cells had lost the plasmid. These experiments demonstrate that *sacB* is a suitable counterselection marker in D. magneticus.

### Construction of a Δ*upp* strain by markerless deletion.

**FIG 3:**
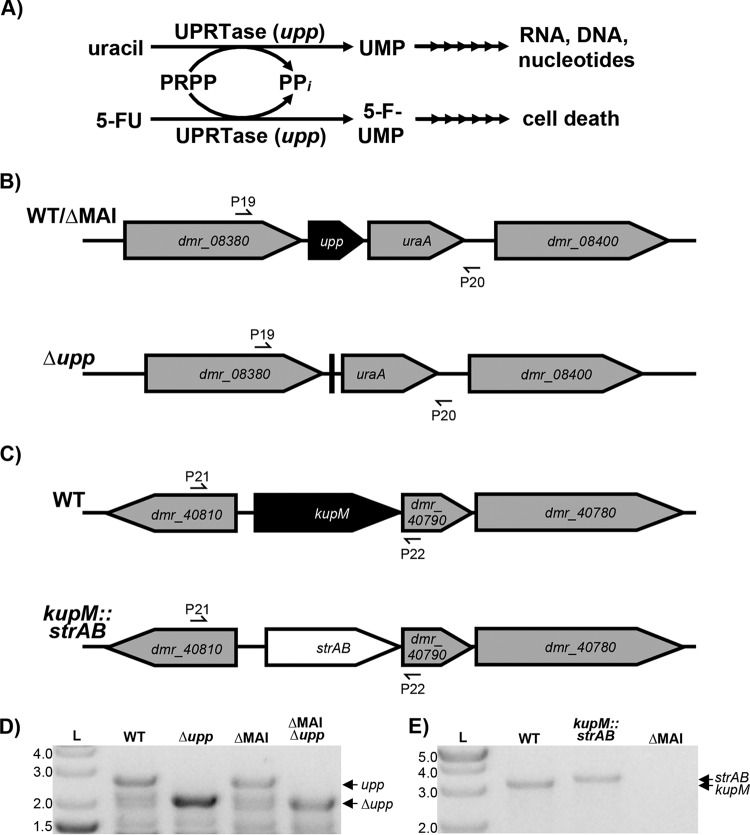
(A) The *upp* gene encodes UPRTase, which is a key enzyme in the uracil salvage pathway. The product of the UPRTase reaction, UMP, is processed by downstream enzymes in pathways for RNA, DNA, and sugar nucleotide synthesis. 5-FU causes cell death by incorporating into this pathway via UPRTase. (B) Schematic of genomic regions of *upp* in the WT or the ΔMAI mutant (top) and the Δ*upp* mutant (bottom). (C) Genomic region of *kup* in WT (top) and *kup*::*strAB* (bottom) strains. Primers used to screen for the correct genotype are indicated with half arrows. (D) Δ*upp* mutants in WT and ΔMAI backgrounds were confirmed by PCR using primers P19/P20 and agarose gel electrophoresis. WT and ΔMAI strains show a band corresponding to the *upp* gene (2,691 bp), while the Δ*upp* mutants have a smaller band corresponding to a markerless deletion of the *upp* gene (2,079 bp). The lower bands are likely nonspecific PCR products. (E) *kupM*::*strAB* genotype confirmation by PCR and agarose gel electrophoresis using primers P21/P22 (WT, 3,069 bp; *kupM*::*strAB*, 3,263 bp; ΔMAI, not applicable [NA]).

**FIG 4:**
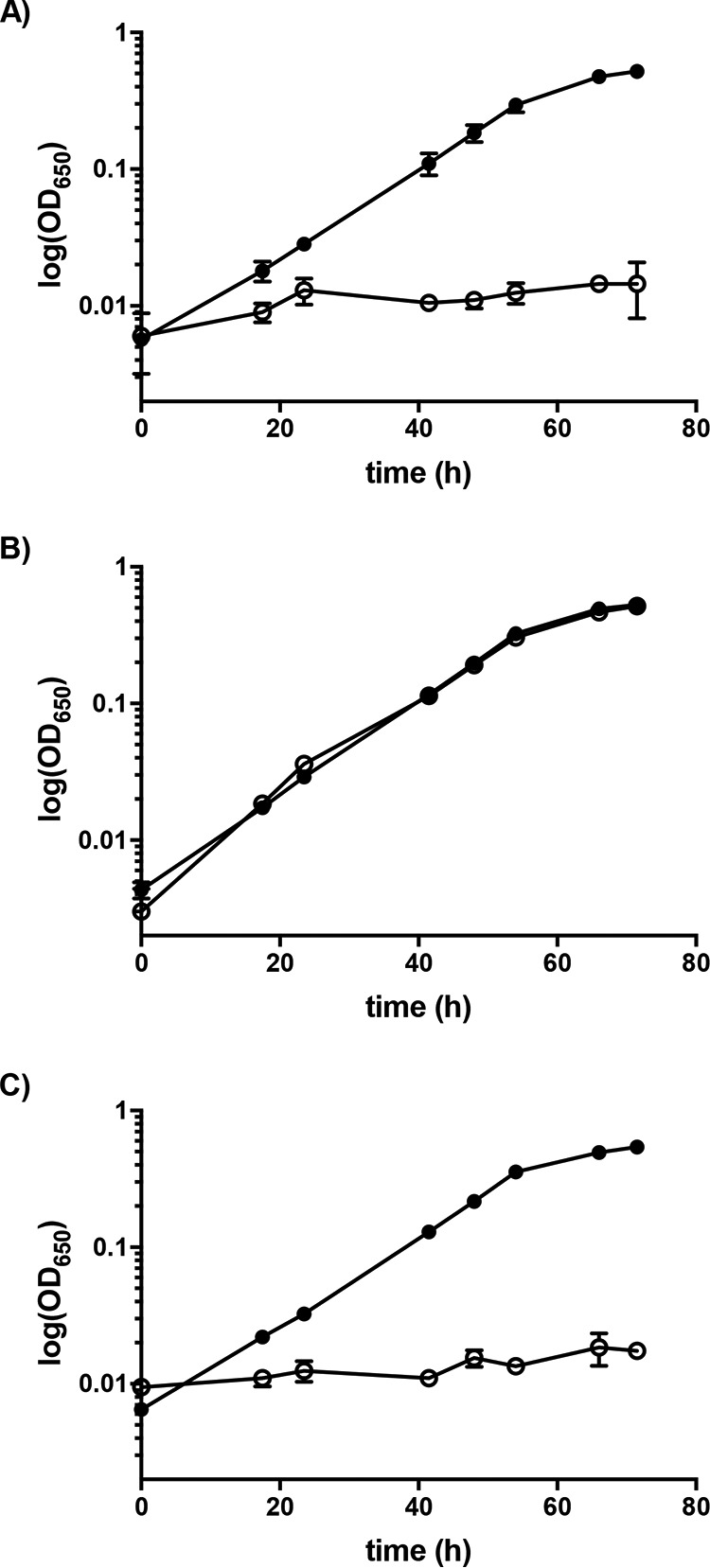
*upp* mutant and complementation phenotype. Growth of the parent strain (ΔMAI) (A), *upp* deletion (ΔMAI Δ*upp*) (B), and complementation of the *upp* deletion (ΔMAI Δ*upp*/*upp^+^*) (C) when grown with 1.25 μg/ml 5-FU (○) or without 5-FU (●). Data presented are averages from 2 to 3 independent cultures; error bars indicate the standard deviations.

To test our replicative deletion method, we chose to target the *upp* gene, the mutation of which has a selectable phenotype. The *upp* gene encodes uracil phosphoribosyltransferase (UPRTase), a key enzyme in the pyrimidine salvage pathway that catalyzes the reaction of uracil with 5-phosphoribosyl-α-1-pyrophosphate (PRPP) to UMP and PP_i_ (35) (Fig. 3A). When given the pyrimidine analog 5-fluorouracil (5-FU), UPRTase catalyzes the production of 5-fluoroxyuridine monophosphate (5-FUMP). 5-FUMP is further metabolized and incorporated into DNA, RNA, and sugar nucleotides resulting in eventual cell death (Fig. 3A) (36, 37). Previous studies have shown that Δ*upp* mutants of D. vulgaris Hildenborough are resistant to 5-FU, while wild-type (WT) cells are effectively killed by the pyrimidine analog (32, 38). The D. magneticus genome has a homolog (*DMR_08390*) to the D. vulgaris Hildenborough *upp* gene that is likely functional, as detected by the sensitivity of D. magneticus to 5-FU (Fig. 3B and 4A). To show that the *upp* gene product confers 5-FU sensitivity and to validate our replicative deletion system, we chose to target the *D. magneticus upp* gene for markerless deletion.

To construct a *upp* deletion vector, a markerless cassette containing the regions upstream and downstream of the *upp* gene were inserted into plasmid pAK914 (Fig. 2B). The resulting plasmid (pAK1126) was transferred to WT D. magneticus by conjugation and single kanamycin-resistant (Kan^r^) colonies were isolated and passaged in growth medium containing no antibiotic. Since *D*. magneticus has interesting features independent of its magnetosomes, the same deletion procedure was also carried out in a nonmagnetic strain (ΔMAI) isolated in our previous genetic studies (20). After the third passage, *upp* mutants that had lost the vector backbone were selected for with 5-FU and sucrose. Compared with those obtained using a control plasmid (pAK914), >20-fold more 5-FU-resistant (5-FU^r^) mutants were generated using pAK1126 at a frequency of approximately 10^−6^. PCR of the region flanking the *upp* gene confirmed that the 5-FU^r^ colonies harboring pAK1126 resulted from a markerless deletion of *upp* (Δ*upp*), while 5-FU^r^ colonies harboring pAK914 were likely the result of point mutations (Fig. 3B and D). Similar to the results obtained for D. vulgaris Hildenborough (32), the Δ*upp* mutant of D. magneticus grew in the presence of 5-FU (Fig. 4B and Table 1). Complementation of the *upp* gene in *trans* restored UPRTase function, and the cells no longer grew with 5-FU (Fig. 2C and 4C and Table 1). These experiments demonstrate that a replicative plasmid can be used to directly edit the D. magneticus genome.

**TABLE 1:** Growth rates and generation times of the parent strain (ΔMAI), Δ*upp* mutant, and *upp* complementation in *trans* with and without treatment with 5-FU

Strain	Growth rate (h^−1^)		Generation time (h)	
	Without 5-FU	With 5-FU	Without 5-FU	With 5-FU
ΔMAI	0.077 ± 0.0017	NA^*a*^	9.1 ± 0.2	NA
ΔMAI Δ*upp*	0.079 ± 0.0017	0.070 ± 0.0040	8.8 ± 0.2	10.0 ± 0.6
ΔMAI Δ*upp*/*upp*^+^	0.076 ± 0.0041	NA	9.1 ± 0.5	NA

a NA, not applicable.

### Construction of a Δ*kup* strain by marker exchange mutagenesis.

Because many genetic mutations do not confer a selectable phenotype, we sought to develop our replicative deletion plasmid for marker exchange mutagenesis. To test this system, we chose to replace a gene with a known phenotype, *kupM* (*DMR_40800*), with a streptomycin-resistance gene cassette (*strAB*). *kupM* is located in the D. magneticus MAI and encodes a functional potassium transporter (20). Mutant alleles in *kupM*, including missense, nonsense, and frameshift mutations, were previously identified in our screen for nonmagnetic mutants (20). These *kupM* mutations resulted in cells that rarely contained electron-dense particles and were unable to turn in a magnetic field, as measured by the coefficient of magnetism (C_mag_) (20).

To mutate *kupM*, we inserted a marker exchange cassette, with regions upstream and downstream of *kupM* flanking *strAB*, into pAK914 (Fig. 2D) to create the deletion plasmid pAK941. Following conjugation, single colonies of D. magneticus harboring pAK941 were isolated by kanamycin selection. After three passages in growth medium without selection, potential mutants were isolated at a frequency of approximately 10^−6^ on plates containing streptomycin and sucrose. Single colonies that were streptomycin resistant (Str^r^) and Suc^r^ were inoculated in liquid medium and screened for Kan^s^. Of the isolates screened (*n* = 48), 20% were Kan^s^ and 4% had the correct genotype (Δ*kupM*::*strAB*) as confirmed by PCR and sequencing (Fig. 3C and E).

**FIG 5:**
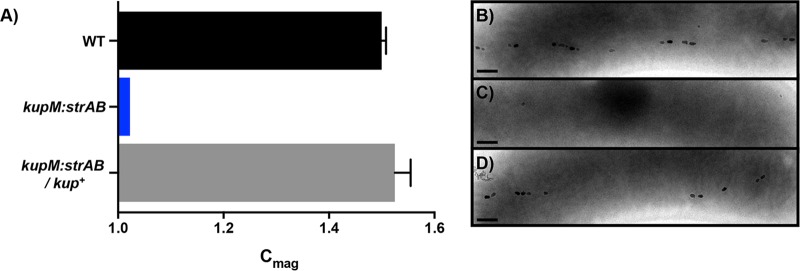
*kupM* mutant and complementation phenotype. C_mag_ values (A) and electron micrographs of WT (B), *kupM*::*strAB* (C), and Δ*kupM*::*strAB/kup^+^* (D) strains. Scale bars, 200 nm. Data presented are averages from 4 independent cultures; error bars indicate the standard deviations.

Similar to the phenotypes previously observed in *kupM* mutants (20), Δ*kupM*::*strAB* cells were severely defective in magnetosome synthesis and turning in a magnetic field (Fig. 5). Although a slight C_mag_ was measured, few cells contained electron-dense particles or magnetosomes. Importantly, the WT phenotype was rescued by expressing *kupM* from a plasmid in the Δ*kupM*::*strAB* mutant (Fig. 5). These results confirm that the replicative deletion plasmid method described here can be used successfully for marker exchange mutagenesis.

## DISCUSSION

In this study, we expand the genetic toolbox for D. magneticus to include a replicative plasmid method for targeted mutagenesis (Fig. 1C). We show the utility of this method for markerless deletion of genes with a selectable phenotype and for marker exchange mutagenesis. Some of the earliest examples of targeted mutagenesis in Gram-negative bacteria used replicative plasmids, similar to the method described here (34, 39). These studies, which predated the application of suicide vectors, relied on plasmid instability by introducing a second plasmid of the same incompatibility group or by limiting nutrients in the growth medium (34, 39).

Because the D. magneticus genetic toolbox has a limited number of plasmids, antibiotic markers, and narrow growth constraints, we used a replicative plasmid and established *sacB* as a counterselection marker to generate and isolate mutants. While *sacB* counterselection was ultimately successful, a large number of false positives were also isolated at the sucrose selection step. Mutations in *sacB* have been found to occur at a high frequency in many bacteria (31, 40–43). Indeed, we found that deletions and mutations in *P_mamA_-sacB* are abundant in the false-positive Suc^r^ Str^r^ isolates (data not shown). Alternative counterselection markers, including *upp*, have been shown to select for fewer false positives (32, 43–45). Since D. magneticus is sensitive to 5-FU only when the *upp* gene is present (Fig. 4), the *upp* mutants generated in this study may be used as the parent strains for future targeted mutagenesis with *upp*, rather than *sacB*, serving as a counterselectable marker. Additionally, the combined use of *upp* and *sacB* for counterselection might reduce the false-positive background that results from the accumulation of mutations in these markers.

The replicative deletion plasmid described here was designed to replace a target gene with an antibiotic resistance marker. As such, the construction of strains with multiple directed mutations will be complicated by the need for additional antibiotic-resistance markers, which are limited in D. magneticus. These limitations may be overcome by removing the chromosomal antibiotic marker in subsequent steps (34, 46, 47). Ultimately, improvements in conjugation efficiency or methods for electroporation with high transformation efficiency are desired. Similar to the ongoing development of genetics in D. vulgaris Hildenborough, the establishment of a suicide vector delivery system in D. magneticus will enable more high-throughput targeted mutagenesis and even the construction of markerless deletion mutants (26, 32).

Overall, we demonstrated the utility of a replicative deletion plasmid to generate targeted mutants of D. magneticus. This method marks a crucial step in developing D. magneticus as a model for the study of anaerobic sulfate reduction and diverse mechanisms of magnetic particle formation by MTB. Both MTB and sulfate-reducing bacteria have been singled out for their role in the global cycling of numerous elements and for potential applications, such as bioremediation (24, 25, 48, 49). D. magneticus, in particular, may be useful in the bioremediation of heavy metals and in the global cycling of iron, since it can form both magnetosomes and other iron-containing organelles (50, 51). Through genetic manipulation of D. magneticus, pathways of elemental cycling and heavy-metal turnover may now be explored. Additionally, genetic manipulation of D. magneticus will further our understanding of magnetosome formation and provide answers to many longstanding questions for the deeply branching MTB. Which proteins regulate and control magnetosome formation? To what extent are lipid membranes involved in forming these crystals? How is the elongated and irregular crystal shape achieved? Finally, in addition to D. magneticus, the method described here may extend to other bacteria that are not amenable to targeted mutagenesis with suicide vectors but are able to accommodate replicative plasmids.

## MATERIALS AND METHODS

### Strains, media, and growth conditions.

The bacterial strains used in this study are listed in Table 2. All E. coli strains were cultured aerobically with continuous shaking at 250 rpm at 37°C in lysogeny broth (LB). D. magneticus strains were grown anaerobically at 30°C in sealed Balch tubes with a N_2_ headspace containing RS-1 growth medium (RGM) that was degassed with N_2_, unless otherwise stated (51). Sodium pyruvate (10 mM) was used as an electron donor with fumaric acid disodium (10 mM) as the terminal electron acceptor. RGM was buffered with HEPES, and the pH was adjusted to 6.7 with NaOH (20). Before inoculating with cells, RGM was supplemented with 0.8% (vol/vol) Wolfe's vitamins, 100 μM ferric malate, and 285 μM cysteine-HCl (51). Solid agar plates were prepared by adding 1.5% agar (wt/vol) to LB and 1% agar (wt/vol) to RGM. Vitamins (0.8% [vol/vol]), ferric malate (20 μM), and cysteine (285 μM), as well as antibiotics and selective agents, were added to the molten RGM agar as needed. For D. magneticus, all plating steps were carried out aerobically, and the bacteria were transferred to an anaerobic jar and incubated at 30°C for 10 to 14 days, as described previously (20). The antibiotics and selective agents used are as follows: kanamycin (50 μg/ml for E. coli strains, 125 μg/ml for D. magneticus strains), streptomycin (50 μg/ml for E. coli and D. magneticus strains), diaminopimelic acid (300 μM for E. coli WM3064), 5-FU (2.5 μg/ml for D. magneticus strains), and sucrose (1% for D. magneticus strains).

**TABLE 2:** Bacterial strains and plasmids used in this study

Strain or plasmid	Genotype or relevant characteristics	Reference or source
Strains		
E. coli		
DH5α λpir	Cloning strain	Lab strain
WM3064	Conjugation strain; DAP auxotroph used for plasmid transfer	Lab strain
D. magneticus		
AK80	Nonmotile mutant of D. magneticus strain RS-1, referred to as wild type	51
AK201	ΔMAI	20
AK267	ΔMAI Δ*upp*	This study
AK268	Δ*upp*	This study
AK270	Δ*kupM*::*strAB*	This study
Plasmids		
pBMK7	Conjugative vector with pBG1 and pMB1 replicons; Kan^r^	53
pBMC7	Conjugative vector with pBG1 and pMB1 replicons; Cm^r^	53
pBMS6	Cloning vector; source of *strAB*; Str^r^	53
pLR6	pBMK7 with *P_mamA_* in HindIII-SalI; source of *P_npt_*; Kan^r^	20
pLR41	pLR6 with *P_mamA_-kupM* in SalI; Kan^r^	20
pAK0	Cloning vector, source of *sacB*; Kan^r^	10
pAK914	pLR6 with *sacB* in SalI-XbaI; Kan^r^	This study
pAK920	pBMC7 with *P_npt_-strAB* inserted into SacI site; Cm^r^ Str^r^	This study
pAK941	pAK914 with cassette of 1,064 bp upstream and 1,057 bp downstream of *kupM* flanking *P_npt_-strAB* in XbaI; Kan^r^ Str^r^	This study
pAK1126	pAK914 with cassette of 991 bp upstream and 1,012 bp downstream of *upp* in XbaI-SacI; Kan^r^	This study
pAK1127	pAK914 with *P_upp_-upp* in BamHI-SacI; Kan^r^	This study

### Plasmids and cloning.

All plasmids used in this work are listed in Table 2. All cloning was performed in E. coli DH5α λpir using the Gibson method (52) or restriction enzyme ligation. For PCR amplification, KOD (EMD Millipore, Germany) and GoTaq (Promega, USA) DNA polymerases were used with the primers listed in Table 3. All upstream and downstream homology regions were amplified from D. magneticus genomic DNA. *strAB* and *P_npt_* were amplified from pBMS6 and pLR6, respectively, and subcloned into pBMC7 to make pAK920, which served as the template for amplifying *P_npt_-strAB* for the deletion vectors. *sacB* was amplified from pAK0 and inserted into pLR6 digested with SalI and XbaI to create pAK914. To construct a plasmid for the targeted deletion of *upp* (*DMR_08390*), 991 bp upstream and 1,012 bp downstream of *upp* were amplified and inserted into pAK914 digested with XbaI and SacI using a 3-piece Gibson assembly. To create the *upp* complementation plasmid, pAK914 was digested with BamHI and SacI, and the *upp* gene, with its promoter, was PCR amplified from D. magneticus genomic DNA. To construct pAK941 for marker exchange mutagenesis of *kupM*, a cassette of a 1,064-bp upstream region and 1,057-bp downstream region flanking *P_npt_-strAB* was assembled using Gibson cloning. The cassette was amplified and inserted into pAK914 digested with XbaI using a two-piece Gibson assembly.

**TABLE 3:** Primers used in this study

Name	Sequence from 5′ end	Description^*a*^
P1	AAGCCAAGAAAAACGTCGCCAACGTCGACATGAACATCAAAAAGTTTGCA	F *sacB* for pAK914
P2	GCTCGGTACCCGGGGATCCTCTAGAGGCCAATAGGATATCGGCATTT	R *sacB* for pAK914
P3	CGACTCTAGAGGATCCCCGGGTACCGTAGCTTCACGCTGCCGCAAG	F *P_npt_* for pAK920
P4	CCCGAATGTGCATGCGAAACGATCCTCATCCTGTC	R *P_npt_* for pAK920
P5	AGGATCGTTTCGCATGCACATTCGGGATATTTCTCTA	F *strAB* for pAK920
P6	TAATACGACTCACTATAGGGAATTCGCCCAGGGGATAGGAGAAGTC	R *strAB* for pAK920
P7	AAATGCCGATATCCTATTGGCCTCTAGAGAGATCGCGAAGCAGAGC	F *kupM* upstream for pAK941
P8	TGCGGCAGCGTGAAGCTACGGTACCGCCGTAATGCGTCAGAAAGT	R *kupM* upstream for pAK941
P9	CTTCTCCTATCCCCTGGGCGAATTCAGCCGGGTCATGGAAGTC	F *kupM* downstream for pAK941
P10	CGAGCTCGGTACCCGGGGATCCTCTAGAGGCCAGGGAATGGAGTTT	R *kupM* downstream for pAK941
P11	GGTACCGTAGCTTCACGCTGCCGCA	F *P_npt_-strAB* for pAK941
P12	GAATTCGCCCAGGGGATAGGAGAAGTCGCT	R *P_npt_-strAB* for pAK941
P13	GCCGATATCCTATTGGCCTCTAGAGCCTCCCAGATCGACCAGTC	F *upp* upstream for pAK1126
P14	CTATTTGGTGCCGGATCCCATGGACGCGCTCCTGGG	R *upp* upstream for pAK1126
P15	AGCGCGTCCATGGGATCCGGCACCAAATAGGGGG	F *upp* downstream for pAK1126
P16	CGACTCACTATAGGGAATTCGAGCTCGCCAGGCAGACGGCGGTG	R *upp* downstream for pAK1126
P17	GCCGATATCCTATTGGCCTCTAGAGAAGCTCGCCGAAAAGACC	F *P_upp_-upp* for pAK1127
P18	CGACTCACTATAGGGAATTCGATGAAGGCGAACGAGGAAC	R *P_upp_-upp* for pAK1127
P19	GCCCGCATTGAGGACGTG	To check *upp* deletion
P20	CAGCGCCCCGAGCTTGCC	To check *upp* deletion
P21	CGTCAGCAGGCAAACGG	To check *kupM* deletion
P22	ACCGTTGTCTCCCATGTCTC	To check *kupM* deletion

a F, forward; R, reverse.

### *upp* and *kup* mutant generation and complementation.

Replicative deletion plasmids were transformed into E. coli WM3064 by heat shock and transferred to D. magneticus by conjugation, as described previously (20). Single colonies of Kan^r^ D. magneticus were isolated and inoculated in RGM containing no antibiotic. Cultures were passaged and, after the third passage, approximately 2 × 10^8^ cells were spread on 1% agar RGM plates containing either 50 μg/ml streptomycin and 1% sucrose or 2.5 μg/ml 5-FU and 1% sucrose. 5-FU^r^ Suc^r^ and Str^r^ Suc^r^ colonies harboring plasmids pAK1126 and pAK941, respectively, were recovered at a frequency of approximately 10^−6^. Single colonies were screened for Kan^s^ and by PCR using the primers listed in Table 3. Successful *upp* and *kup* mutants were confirmed by Sanger sequencing. The expression plasmids for the complementation of Δ*kup*::*strAB* and Δ*upp*, as well as empty vectors for controls, were transferred to D. magneticus strains as described above. Transconjugants were inoculated in RGM containing kanamycin to maintain the plasmids.

### Mutant phenotype and complementation analyses.

The growth and coefficient of magnetism (C_mag_) of D. magneticus strains were measured in a Spec20 spectrophotometer at an optical density of 650 nm (OD_650_), as described previously (10, 51). For *upp* mutant and complementation analyses, RGM was supplemented with 5-FU (1.25 μg/ml in 0.01% dimethyl sulfoxide [DMSO]) or DMSO (0.01%), and the growth was measured for WT and Δ*upp* strains with an empty vector (pAK914) and for the Δ*upp* strain with the complementation plasmid pAK1127. For *kup* mutant and complementation analyses, the C_mag_ was measured by placing a large bar magnet parallel or perpendicular to the sample to measure the maximum or minimum absorbance, respectively, as the D. magneticus strains rotate 90° with the magnetic field. The ratio of maximum to minimum absorbances was calculated as the C_mag_ (10). Whole-cell transmission electron microscopy (TEM) was performed as previously described (51). The C_mag_ calculations and TEM were performed for WT D. magneticus with an empty vector (pBMK7) and the Δ*kup*::*strAB* strain with an empty vector (pBMK7) or complementation plasmid (pLR41). For all growth measurements, C_mag_ measurements, and TEM, the cells harboring the plasmids were maintained with 125 μg/ml kanamycin.

## References

[1] Bellini S 2009 On a unique behavior of freshwater bacteria. Chin J Oceanol Limnol 27:3. doi:10.1007/s00343-009-0003-5.

[2] Blakemore R 1975 Magnetotactic bacteria. Science 190:377–379. doi:10.1126/science.170679. 170679

[3] Uebe R, Schüler D 2016 Magnetosome biogenesis in magnetotactic bacteria. Nat Rev Microbiol 14:621–637. doi:10.1038/nrmicro.2016.99. 27620945

[4] Bazylinski DA, Frankel RB, Jannasch HW 1988 Anaerobic magnetite production by a marine, magnetotactic bacterium. Nature 334:518–519. doi:10.1038/334518a0.

[5] Blakemore RP, Maratea D, Wolfe RS 1979 Isolation and pure culture of a freshwater magnetic spirillum in chemically defined medium. J Bacteriol 140:720–729. 500569 10.1128/jb.140.2.720-729.1979 PMC216702

[6] Matsunaga T, Sakaguchi T, Tadakoro F 1991 Magnetite formation by a magnetic bacterium capable of growing aerobically. Appl Microbiol Biotechnol 35:651–655. doi:10.1007/BF00169632.

[7] Schüler D, Köhler M 1992 The isolation of a new magnetic spirillum. Zentralbl Mikrobiol 147:150–151. doi:10.1016/S0232-4393(11)80377-X.

[8] Balkwill DL, Maratea D, Blakemore RP 1980 Ultrastructure of a magnetotactic spirillum. J Bacteriol 141:1399–1408. 6245069 10.1128/jb.141.3.1399-1408.1980 PMC293838

[9] Gorby YA, Beveridge TJ, Blakemore RP 1988 Characterization of the bacterial magnetosome membrane. J Bacteriol 170:834–841. doi:10.1128/jb.170.2.834-841.1988. 3123464 PMC210730

[10] Komeili A, Vali H, Beveridge TJ, Newman DK 2004 Magnetosome vesicles are present before magnetite formation, and MamA is required for their activation. Proc Natl Acad Sci U S A 101:3839–3844. doi:10.1073/pnas.0400391101. 15004275 PMC374331

[11] Komeili A 2012 Molecular mechanisms of compartmentalization and biomineralization in magnetotactic bacteria. FEMS Microbiol Rev 36:232–255. doi:10.1111/j.1574-6976.2011.00315.x. 22092030 PMC3540109

[12] Pósfai M, Lefèvre C, Trubitsyn D, Bazylinski DA, Frankel R 2013 Phylogenetic significance of composition and crystal morphology of magnetosome minerals. Front Microbiol 4:344. doi:10.3389/fmicb.2013.00344. 24324461 PMC3840360

[13] Lefèvre CT, Bazylinski DA 2013 Ecology, diversity, and evolution of magnetotactic bacteria. Microbiol Mol Biol Rev 77:497–526. doi:10.1128/MMBR.00021-13. 24006473 PMC3811606

[14] Lin W, Zhang W, Zhao X, Roberts AP, Paterson GA, Bazylinski DA, Pan Y 2018 Genomic expansion of magnetotactic bacteria reveals an early common origin of magnetotaxis with lineage-specific evolution. ISME J 12:1508–1519. doi:10.1038/s41396-018-0098-9. 29581530 PMC5955933

[15] Kolinko I, Lohße A, Borg S, Raschdorf O, Jogler C, Tu Q, Pósfai M, Tompa É, Plitzko JM, Brachmann A, Wanner G, Müller R, Zhang Y, Schüler D 2014 Biosynthesis of magnetic nanostructures in a foreign organism by transfer of bacterial magnetosome gene clusters. Nat Nanotechnol 9:193–197. doi:10.1038/nnano.2014.13. 24561353

[16] Murat D, Quinlan A, Vali H, Komeili A 2010 Comprehensive genetic dissection of the magnetosome gene island reveals the step-wise assembly of a prokaryotic organelle. Proc Natl Acad Sci U S A 107:5593–5598. doi:10.1073/pnas.0914439107. 20212111 PMC2851823

[17] Murat D, Falahati V, Bertinetti L, Csencsits R, Körnig A, Downing K, Faivre D, Komeili A 2012 The magnetosome membrane protein, MmsF, is a major regulator of magnetite biomineralization in *Magnetospirillum magneticum* AMB-1. Mol Microbiol 85:684–699. doi:10.1111/j.1365-2958.2012.08132.x. 22716969 PMC3570065

[18] Lefèvre CT, Trubitsyn D, Abreu F, Kolinko S, Jogler C, de Almeida LGP, de Vasconcelos ATR, Kube M, Reinhardt R, Lins U, Pignol D, Schüler D, Bazylinski DA, Ginet N 2013 Comparative genomic analysis of magnetotactic bacteria from the *Deltaproteobacteria* provides new insights into magnetite and greigite magnetosome genes required for magnetotaxis. Environ Microbiol 15:2712–2735. doi:10.1111/1462-2920.12128. 23607663

[19] Lin W, Deng A, Wang Z, Li Y, Wen T, Wu L-F, Wu M, Pan Y 2014 Genomic insights into the uncultured genus “*Candidatus* Magnetobacterium” in the phylum *Nitrospirae*. ISME J 8:2463–2477. doi:10.1038/ismej.2014.94. 24914800 PMC4260714

[20] Rahn-Lee L, Byrne ME, Zhang M, Sage DL, Glenn DR, Milbourne T, Walsworth RL, Vali H, Komeili A 2015 A genetic strategy for probing the functional diversity of magnetosome formation. PLoS Genet 11:e1004811. doi:10.1371/journal.pgen.1004811. 25569806 PMC4287615

[21] Sakaguchi T, Arakaki A, Matsunaga T 2002 *Desulfovibrio magneticus* sp. nov., a novel sulfate-reducing bacterium that produces intracellular single-domain-sized magnetite particles. Int J Syst Evol Microbiol 52:215–221. doi:10.1099/00207713-52-1-215. 11837306

[22] Sakaguchi T, Burgess JG, Matsunaga T 1993 Magnetite formation by a sulphate-reducing bacterium. Nature 365:47–49. doi:10.1038/365047a0.

[23] Nakazawa H, Arakaki A, Narita-Yamada S, Yashiro I, Jinno K, Aoki N, Tsuruyama A, Okamura Y, Tanikawa S, Fujita N, Takeyama H, Matsunaga T 2009 Whole genome sequence of *Desulfovibrio magneticus* strain RS-1 revealed common gene clusters in magnetotactic bacteria. Genome Res 19:1801–1808. doi:10.1101/gr.088906.108. 19675025 PMC2765288

[24] Barton LL, Fauque GD 2009 Chapter 2. Biochemistry, physiology and biotechnology of sulfate-reducing bacteria, p 41–98. *In* Laskin A, Gadd G, Sariaslani S (ed), Advances in applied microbiology, vol 68 Academic Press, Cambridge, MA. 10.1016/S0065-2164(09)01202-7 19426853

[25] Heidelberg JF, Seshadri R, Haveman SA, Hemme CL, Paulsen IT, Kolonay JF, Eisen JA, Ward N, Methe B, Brinkac LM, Daugherty SC, Deboy RT, Dodson RJ, Durkin AS, Madupu R, Nelson WC, Sullivan SA, Fouts D, Haft DH, Selengut J, Peterson JD, Davidsen TM, Zafar N, Zhou L, Radune D, Dimitrov G, Hance M, Tran K, Khouri H, Gill J, Utterback TR, Feldblyum TV, Wall JD, Voordouw G, Fraser CM 2004 The genome sequence of the anaerobic, sulfate-reducing bacterium *Desulfovibrio vulgaris* Hildenborough. Nat Biotechnol 22:554–559. doi:10.1038/nbt959. 15077118

[26] Keller KL, Wall JD 2011 Genetics and molecular biology of the electron flow for sulfate respiration in *Desulfovibrio*. Front Microbiol 2:135. doi:10.3389/fmicb.2011.00135. 21747813 PMC3129016

[27] Wall JD, Hemme CL, Rapp-Giles B, Ringbauer JA, Casalot L, Giblin T 2003 Genes and genetic manipulations of *Desulfovibrio*, p 85–98. *In* Ljungdahl LG, Adams MW, Barton LL, Ferry JG, Johnson MK (ed), Biochemistry and physiology of anaerobic bacteria. Springer, New York, NY.

[28] Broco M, Rousset M, Oliveira S, Rodrigues-Pousada C 2005 Deletion of flavoredoxin gene in *Desulfovibrio gigas* reveals its participation in thiosulfate reduction. FEBS Lett 579:4803–4807. doi:10.1016/j.febslet.2005.07.044. 16099456

[29] Parks JM, Johs A, Podar M, Bridou R, Hurt RA, Smith SD, Tomanicek SJ, Qian Y, Brown SD, Brandt CC, Palumbo AV, Smith JC, Wall JD, Elias DA, Liang L 2013 The genetic basis for bacterial mercury methylation. Science 339:1332–1335. doi:10.1126/science.1230667. 23393089

[30] Rousset M, Dermoun Z, Chippaux M, Bélaich JP 1991 Marker exchange mutagenesis of the *hydN* genes in *Desulfovibrio fructosovorans*. Mol Microbiol 5:1735–1740. doi:10.1111/j.1365-2958.1991.tb01922.x. 1943706

[31] Fu R, Voordouw G 1997 Targeted gene-replacement mutagenesis of *dcrA*, encoding an oxygen sensor of the sulfate-reducing bacterium *Desulfovibrio vulgaris* Hildenborough. Microbiology 143:1815–1826. doi:10.1099/00221287-143-6-1815. 9202456

[32] Keller KL, Bender KS, Wall JD 2009 Development of a markerless genetic exchange system for *Desulfovibrio vulgaris* Hildenborough and its use in generating a strain with increased transformation efficiency. Appl Environ Microbiol 75:7682–7691. doi:10.1128/AEM.01839-09. 19837844 PMC2794091

[33] Gay P, Coq DL, Steinmetz M, Ferrari E, Hoch JA 1983 Cloning structural gene *sacB*, which codes for exoenzyme levansucrase of *Bacillus subtilis*: expression of the gene in *Escherichia coli*. J Bacteriol 153:1424–1431. 6402497 10.1128/jb.153.3.1424-1431.1983 PMC221793

[34] Ried JL, Collmer A 1987 An *nptI-sacB-sacR* cartridge for constructing directed, unmarked mutations in Gram-negative bacteria by marker exchange-eviction mutagenesis. Gene 57:239–246. doi:10.1016/0378-1119(87)90127-2. 3319780

[35] Neuhard J 1983 Utilization of preformed pyrimidine bases and nucleosides, p 95–148. *In* Munch-Petersen A (ed), Metabolism of nucleotides, nucleosides and nucleobases in microorganisms. Academic Press, New York, NY.

[36] Singh V, Brecik M, Mukherjee R, Evans JC, Svetlíková Z, Blaško J, Surade S, Blackburn J, Warner DF, Mikušová K, Mizrahi V 2015 The complex mechanism of antimycobacterial action of 5-fluorouracil. Chem Biol 22:63–75. doi:10.1016/j.chembiol.2014.11.006. 25544046

[37] Cohen SS, Flaks JG, Barner HD, Loeb MR, Lichtenstein J 1958 The mode of action of 5-fluorouracil and its derivatives. Proc Natl Acad Sci U S A 44:1004–1012. 16590300 10.1073/pnas.44.10.1004 PMC528686

[38] Bender KS, Cheyen H, Wall JD 2006 Analysing the metabolic capabilities of *Desulfovibrio* species through genetic manipulation. Biotechnol Genet Eng Rev 23:157–174. doi:10.1080/02648725.2006.10648083. 22530507

[39] Ruvkun GB, Ausubel FM 1981 A general method for site-directed mutagenesis in prokaryotes. Nature 289:85–88. doi:10.1038/289085a0. 6256652

[40] Bloor AE, Cranenburgh RM 2006 An efficient method of selectable marker gene excision by Xer recombination for gene replacement in bacterial chromosomes. Appl Environ Microbiol 72:2520–2525. doi:10.1128/AEM.72.4.2520-2525.2006. 16597952 PMC1449051

[41] Cai YP, Wolk CP 1990 Use of a conditionally lethal gene in *Anabaena* sp. strain PCC 7120 to select for double recombinants and to entrap insertion sequences. J Bacteriol 172:3138–3145. doi:10.1128/jb.172.6.3138-3145.1990. 2160938 PMC209118

[42] Kaniga K, Delor I, Cornelis GR 1991 A wide-host-range suicide vector for improving reverse genetics in Gram-negative bacteria: inactivation of the *blaA* gene of *Yersinia enterocolitica*. Gene 109:137–141. doi:10.1016/0378-1119(91)90599-7. 1756974

[43] Ma W, Wang X, Mao Y, Wang Z, Chen T, Zhao X 2015 Development of a markerless gene replacement system in *Corynebacterium glutamicum* using *upp* as a counter-selection marker. Biotechnol Lett 37:609–617. doi:10.1007/s10529-014-1718-8. 25376333

[44] Graf N, Altenbuchner J 2011 Development of a method for markerless gene deletion in *Pseudomonas putida*. Appl Environ Microbiol 77:5549–5552. doi:10.1128/AEM.05055-11. 21666018 PMC3147467

[45] Wang Y, Zhang C, Gong T, Zuo Z, Zhao F, Fan X, Yang C, Song C 2015 An *upp*-based markerless gene replacement method for genome reduction and metabolic pathway engineering in *Pseudomonas mendocina* NK-01 and *Pseudomonas putida* KT2440. J Microbiol Methods 113:27–33. doi:10.1016/j.mimet.2015.03.022. 25828098

[46] Fabret C, Dusko Ehrlich S, Noirot P 2002 A new mutation delivery system for genome-scale approaches in *Bacillus subtilis*. Mol Microbiol 46:25–36. doi:10.1046/j.1365-2958.2002.03140.x. 12366828

[47] Huang LC, Wood EA, Cox MM 1997 Convenient and reversible site-specific targeting of exogenous DNA into a bacterial chromosome by use of the FLP recombinase: the FLIRT system. J Bacteriol 179:6076–6083. doi:10.1128/jb.179.19.6076-6083.1997. 9324255 PMC179511

[48] Chen AP, Berounsky VM, Chan MK, Blackford MG, Cady C, Moskowitz BM, Kraal P, Lima EA, Kopp RE, Lumpkin GR, Weiss BP, Hesse P, Vella NGF 2014 Magnetic properties of uncultivated magnetotactic bacteria and their contribution to a stratified estuary iron cycle. Nat Commun 5:4797. doi:10.1038/ncomms5797. 25175931

[49] Lin W, Bazylinski DA, Xiao T, Wu L-F, Pan Y 2014 Life with compass: diversity and biogeography of magnetotactic bacteria. Environ Microbiol 16:2646–2658. doi:10.1111/1462-2920.12313. 24148107

[50] Arakaki A, Takeyama H, Tanaka T, Matsunaga T 2002 Cadmium recovery by a sulfate-reducing magnetotactic bacterium, *Desulfovibrio magneticus* RS-1, using magnetic separation. Appl Biochem Biotechnol 98–100:833–840. 10.1385/abab:98-100:1-9:833 12018305

[51] Byrne ME, Ball DA, Guerquin-Kern J-L, Rouiller I, Wu T-D, Downing KH, Vali H, Komeili A 2010 *Desulfovibrio magneticus* RS-1 contains an iron- and phosphorus-rich organelle distinct from its bullet-shaped magnetosomes. Proc Natl Acad Sci U S A 107:12263–12268. doi:10.1073/pnas.1001290107. 20566879 PMC2901430

[52] Gibson DG, Young L, Chuang RY, Venter JC, Hutchison CA III, Smith HO 2009 Enzymatic assembly of DNA molecules up to several hundred kilobases. Nat Methods 6:343–345. doi:10.1038/nmeth.1318. 19363495

[53] Rousset M, Casalot L, Rapp-Giles BJ, Dermoun Z, de Philip P, Bélaich J-P, Wall JD 1998 New shuttle vectors for the introduction of cloned DNA in *Desulfovibrio*. Plasmid 39:114–122. doi:10.1006/plas.1997.1321. 9514705

